# Study of the SCL-90 Scale and Changes in the Chinese Norms

**DOI:** 10.3389/fpsyt.2020.524395

**Published:** 2021-01-27

**Authors:** Weimin Dang, Yajuan Xu, Jun Ji, Ke Wang, Songtao Zhao, Bin Yu, Jinming Liu, Chaonan Feng, Haokui Yu, Wenqiang Wang, Xin Yu, Wentian Dong, Yantao Ma

**Affiliations:** ^1^NHC Key Laboratory of Mental Health (Peking University), National Clinical Research Center for Mental Disorders (Peking University Sixth Hospital), Peking University Sixth Hospital, Peking University Institute of Mental Health, Beijing, China; ^2^Department of Psychiatry, Xiamen Xianyue Hospital, Xiamen, China; ^3^College of Computer Science and Technology, Qingdao University, Qingdao, China; ^4^Beijing Wanling Pangu Science and Technology Ltd., Beijing, China; ^5^Medical College, Qingdao University, Qingdao, China; ^6^Psychiatric Department, Qingdao Municipal Hospital, Qingdao, China; ^7^Linyi Mental Health Center, Linyi, China

**Keywords:** SCL-90, Chinese norms, critical value, scores of each factor, norm changes

## Abstract

**Objective:** This study aimed to investigate the Chinese norms for the Symptom Checklist 90 (SCL-90) scale and its application.

**Methods:** In total, 7,489 adults from Tianjin and Qingdao in China were included. Their data were compared with the norm data of 1,388 people published by Jin et al., the combined norms published by Tang et al., the data of 2,808 adults published by Chen and Li, and the data of 1,890 adults from Tong in China.

**Results:** In five different periods, notable changes were observed in each factor of the SCL-90 that significantly differed from the previous norms. The scores of each factor showed an increasing annual trend. Compulsion consistently obtained the highest scores, and phobia consistently obtained the lowest scores. The scores tended to decrease from compulsion to anxiety, and psychosis scored lower than paranoia. There was a significant difference in the detection rate between the critical screening value of two points and the standard score. Using the standard score as the critical value, the detection rate ranged between 13 and 16% and was relatively concentrated. Using two points as the critical value, the detection rate ranged between 38 and 50%.

**Conclusion:** The usual model in China is not consistent with social development. Using two points as the critical value is no longer suitable for the SCL-90. New Chinese norms and measurement standards should be developed. The mean value plus one standard deviation could be used as the new measurement standard.

## Introduction

The Symptom Checklist 90 (SCL-90) is a psychosomatic screening scale proposed by Derogatis that is widely used in China and elsewhere (1). The SCL-90 can be used to distinguish between patients with and without psychosomatic diseases and has good reliability and validity (2, 3). However, the SCL-90 lacks widely accepted norms (4). The SCL-90 scale currently used in China was translated by Wang (5). For the translated version of the scale, Jin and Wu published a set of data of Chinese norms in 1986 that were based on data from 1,388 patients (6). Subsequently, many application studies have been performed in China, and these studies mainly included surveys of the general population and studies concerning the obstacles to large-scale screening. In 1999, Wang et al. (7) proposed a norm suitable for Chinese middle school students. In 1999, Tang et al. (8) proposed a combined norm based on 47,354 people by integrating the Chinese literature. Chen and Li (9) examined a sample of 2,808 people in 2003 and discussed the combined norm.

Regarding its application, an important problem with the scale is its relatively lack of time-effectiveness, which is not only a concern in populations with mental health concerns but also results in varying levels of performance at different times and in populations with different cultural backgrounds. Many studies concerning this issue were published as much as a decade ago. The norm for Wang's translation, which is currently used in China, was proposed in 1986 and is more than 30 years old. Since the Chinese economic reform policy was implemented in the 1980s, significant social changes have occurred in China, making it necessary to update the norms of the SCL-90. This study was performed to compare the differences between data from a current sample population and previously reported sample population data or norms and study the changes in different factors of the SCL-90.

## Materials and Methods

### Ethics

These centers do not have ethics committees. Their role is to perform checkups for consumers but not patients, and they have no diagnostic responsibility. However, there is an electronic consent form (written in Chinese) that each subject (the consumer in the context of these physical examination centers) signs before the administration of the psychological tests. In section 10.1 of the electronic consent form, the following is presented: “The owner of this testing system has the right to perform analyses of non-sensitive data.”

### Subjects

The subjects were 7,489 people aged 20–45 years who were selected from 10 medical centers, including four commercial medical examination centers in Tianjin five commercial medical examination centers and one public hospital medical examination center in Qingdao China, from January to August 2019. These visitors all received health checkups and had no clear psychiatric problems. A health checkup was performed by a psychological counselor.

### Instruments

The version of the SCL-90 translated by Wang was used (5).

### Statistical Analysis

The SCL-90 data of 7,489 persons evaluated in 2019 were compared with previously published norms. The data used for the comparisons were obtained from previously published Chinese studies and consisted of the following: (1) norm data of 1,388 people published by Jin et al. (6); (2) combined norm data of 47,354 people summarized in the literature by Tang et al. (8) in 1999; (3) data of 2,808 people from Hangzhou, China reported by Chen and Li (9) in 1999; and (4) a nationwide sample of 1,890 people in China presented by Tong in 2006 (4). Python software was used for the data analysis. Due to the large sample size, a *z*-test was used. A *P*-value < 0.05 was considered statistically significant.

## Results

### Comparative Analysis of Data From Five Studies

We compared the data of 7,489 people in 2019 with the 1986 norms, the data of 47,354 people published by Tang in 1999, the data of 2,808 people published by Chen and Li (9) and the data published by Tong in 2006. We found that the scores of all factors in the 2019 Chinese data were significantly higher than those in previous years and that the data were more concentrated (see Tables 1–3).

**Table 1:** Comparison between the 2019 data and Chinese norms in 1986.

**Subscales**	**1986** (***n*** **=** **1,388**)		**2019** (***n*** **=** **7,489**)		***Z***
	**M**	**SD**	**M**	**SD**	
Somatization	1.37	0.48	1.961	0.639	−39.83^***^
Obsessive compulsive	1.62	0.58	2.396	0.657	−44.80^***^
Interpersonal sensitivity	1.65	0.51	2.105	0.687	−28.74^***^
Depression	1.5	0.59	2.018	0.639	−29.65^***^
Anxiety	1.39	0.43	1.942	0.607	−40.87^***^
Hostility	1.48	0.56	2.044	0.661	−33.47^***^
Phobic anxiety	1.23	0.41	1.514	0.544	−22.38^***^
Paranoid ideation	1.43	0.57	1.936	0.653	−29.65^***^
Psychoticism	1.29	0.42	1.744	0.594	−34.41^***^

* *p < 0.05*;

** *p < 0.01*;

*** *p < 0.001*;

*notably, the same applies below*.

**Table 2:** Comparison of the data published by Tang et al. (8), Chen and Li (9) and 2019.

**Subscales**	**Tang (1999;** ***n*** **=** **47,354**)		**Chen** (**1999;** ***n*** **=** **2,808**)		***Z***	
	**M**	**SD**	**M**	**SD**	**Tang 2019**	**Chen 2019**
Somatization	1.48	0.54	1.36	0.39	−61.93^***^	−57.74^***^
Obsessive compulsive	1.83	0.64	1.47	0.45	−69.52^***^	−81.29^***^
Interpersonal sensitivity	1.79	0.65	1.44	0.45	−37.13^***^	−57.20^***^
Depression	1.70	0.65	1.33	0.39	−39.93^***^	−66.00^***^
Anxiety	1.55	0.55	1.30	0.37	−52.59^***^	−64.87^***^
Hostility	1.64	0.63	1.36	0.41	−49.52^***^	−62.96^***^
Phobic anxiety	1.40	0.50	1.17	0.30	−16.98^***^	−40.62^***^
Paranoid ideation	1.69	0.62	1.32	0.42	−30.47^***^	−56.26^***^
Psychoticism	1.53	0.56	1.25	0.34	−29.20^***^	−52.58^***^

* *p < 0.05*;

** *p < 0.01*;

*** *p < 0.001*.

**Table 3:** Comparison between the 2019 and 2006 data.

**Subscales**	**2006** (***n*** **=** **1,890**)		**2019** (***n*** **=** **7,489**)		***Z***
	**M**	**SD**	**M**	**SD**	
Somatization	1.42	0.44	1.961	0.637	−43.09^***^
Obsessive compulsive	1.66	0.52	2.396	0.657	−52.30^***^
Interpersonal sensitivity	1.51	0.49	2.105	0.687	−42.82^***^
Depression	1.50	0.47	2.018	0.639	−39.68^***^
Anxiety	1.34	0.39	1.942	0.607	−52.66^***^
Hostility	1.50	0.51	2.044	0.661	−39.29^***^
Phobic anxiety	1.27	0.39	1.514	0.544	−22.50^***^
Paranoid ideation	1.44	0.47	1.936	0.653	−37.93^***^
Psychoticism	1.33	0.39	1.744	0.594	−37.14^***^

* *p < 0.05*;

** *p < 0.01*;

*** *p < 0.001*.

**Figure 1:**
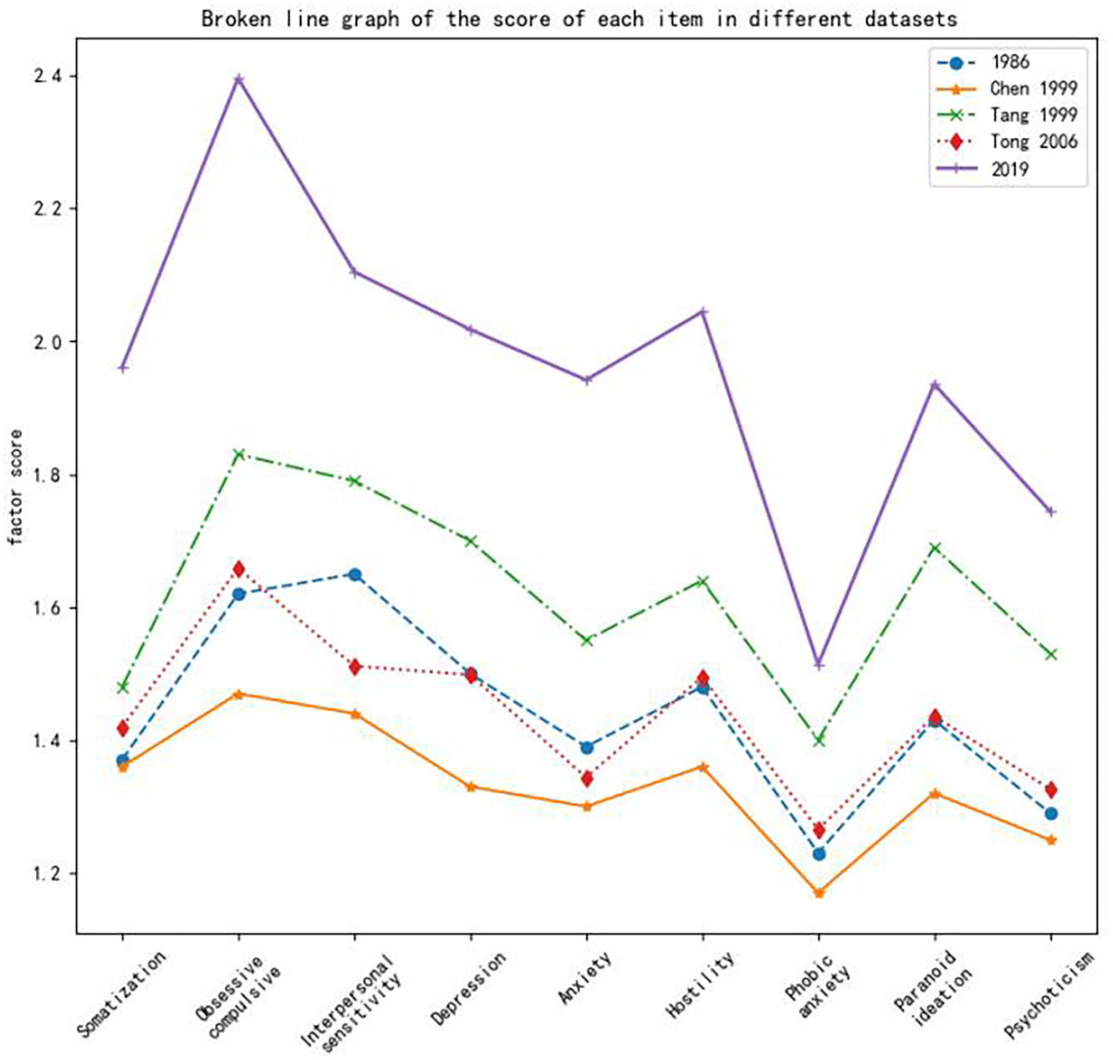
Broken line graph of the score of each item in different datasets.

Figure 1 shows the differences in the scores across the five different sample populations. Figure 1 shows that the Chinese norms in 2006 are close to those in 1986, although there are differences. The scores of each factor increased annually. The comparison between the 2019 data and Tang's 1999 data shows the most obvious changes. The trends in the factor scores across the different sample datasets were generally consistent. All data sets showed that compulsion factors had the highest scores and that phobic anxiety factors had the lowest scores. The scores showed a tendency to decline from compulsion to anxiety, and the scores of psychosis were lower than those of paranoid ideation.

### Different Meanings of Subjective Perception Evaluation and Norm Evaluation

The authors of the studies expressed different opinions regarding the critical value for each dimension of the SCL-90. Some studies used two points as the critical value for each factor for screening purposes (10). Whether this critical value of two points is valid urgently needs to be determined. Therefore, in this experiment, the hypothesis was examined based on the norms of China. The detection rate was calculated based on the mean value in 2019 plus one standard deviation as the critical value and compared with the detection rate calculated based on two points as the critical value. We found significant differences in all factors, except for phobic anxiety. Using the mean score as the critical value, the detection rate ranged between 13 and 16% and was relatively concentrated. However, using two points as the critical value, the detection rate was concentrated between 38 and 50%, except for compulsion (70.09%), phobic anxiety (13.98%) and psychosis (26.21%) (see Table 4).

**Table 4:** Comparison of the subjective perception rating and norm detection rate.

**Subscales**	**Mean (detection rate)**	**two points (detection rate)**	***Z***
Somatization	1,132 (15.12%)	3,034 (40.51%)	26.95^***^
Obsessive compulsive	1,082 (14.45%)	5,249 (70.09%)	55.12^***^
Interpersonal sensitivity	1,046 (13.97%)	3,810 (50.87%)	38.25^***^
Depression	1,223 (16.33%)	3,442 (45.96%)	30.37^***^
Anxiety	1,226 (16.37%)	3,051 (40.74%)	25.14^***^
Hostility	1,028 (13.73%)	3,191 (42.61%)	34.37^***^
Phobic anxiety	1,047 (13.98%)	1,047 (13.98%)	0
Paranoid ideation	1,180 (15.75%)	2,870 (38.32%)	24.28^***^
Psychoticism	1,209 (16.14%)	1,963 (26.21%)	12.71^***^

* *p < 0.05*;

** *p < 0.01*;

*** *p < 0.001*.

## Discussion

The SCL-90 scale is widely used worldwide (1, 11). The SCL-90 is a screening scale and needs to be revised regularly. China is currently using Wang's translated version from 1984 and the norms proposed in 1986, which are currently more than 30 years old. The rapid development of society has inevitably led to psychological changes in the Chinese population. If we continue to use the norms published 30 years ago for research, these data will be inconsistent with the current psychological status of the population. Therefore, this study compared the data of five large sample populations collected over time and concluded that the current population has notable differences from previous populations in several factors. Moreover, the scores of all factors tended to increase annually, which is consistent with the research by Tang, Chen and others. In this general upward trend, the differences between the neurotic and psychopathic factors were particularly prominent in the 2019 study. The neurotic features of the population, such as depression, anxiety, obsessive compulsive, and somatization, were significantly higher than the psychotic symptoms, such as paranoid ideation and psychoticism. The individuals included in this study were young people aged between 20 and 45 years who experienced high levels of pressure, which may have contributed to the high scores of various factors. The scores of each factor increased annually. Thus, it is inadvisable to continue to use the previously established norms, which are not consistent with how Chinese society developed, and new Chinese norms are urgently needed.

In previous studies, the sample sizes used to generate norms were not large and mostly ranged between 1,000 and 3,000 (4, 6, 9). Although the norms proposed by Tang et al. were based on data from 47,354 people, the data were obtained over the course of 7 years (8). However, the sample size in this study was 7,000, ensuring greater accuracy. The individuals included in this study were mostly enrolled at physical examination centers, enabling the inclusion of a large sample population due to the high degrees of mobility and compliance. This method is a feasible approach to obtaining large sample sizes in future studies. Most previous studies were conducted in hospitals. In recent years, studies conducted in physical examination centers have gradually increased (12, 13), but the sample size has remained small. This study can provide more information for the use of norms in physical examination settings.

### The Critical Value

Each item on the SCL-90 is scored on a five-point scale from 1 to 5. One point indicates no symptoms, and two points indicates mild symptoms (14). The authors of the scale did not propose a critical value. Generally, the value of two points is used for screening based on experience rather than the standard score (10–15). In 1999, Tang et al. (8) proposed that the mean value plus one standard deviation should be used as the critical value. This study showed that there is a large difference in the screening results when the mean and two points are used. Using the mean score as the critical value, the detection rate ranged between 13 and 16%, which is reasonable. When the critical value was two points, the detection rate varied from 13 to 70%, which covered a large range. This finding indicates a reduced specificity of detection. Thus, the critical value of two points is no longer suitable as the critical value when the SCL-90 is used for screening purposes. New standards are urgently needed. The mean value plus one standard deviation is a candidate standard.

### Comparison With Norm Data From Other Countries

According to the literature (16), in the US population, the average score of each item of the SCL-90 was lower than that reported here with an average score below 0.5 and scores concentrated in the range from 0.2 to 0.4, except for phobic anxiety (0.13) and psychosis (0.14). The average scores of all items among New Zealand college students ranged between 0.7 and 1.2, except for phobic anxiety (0.28). A study showed (17) that the scores of all items in the German population ranged between 0.29 and 0.5, except for phobic anxiety (0.14) and psychosis (0.18). The scores of obsessive-compulsive symptoms were the highest, and there was a downward trend in the scores from compulsion to anxiety. Other studies (16, 18) have shown that the scores of each item slightly increase as the economy continues to develop in the United States, but the average scores of all items in the British population ranged between 0.4 and 0.6, except for phobic anxiety (0.24) and psychosis (0.27). A study involving college students in Spain (19, 20) showed that the scores of all items ranged between 0.4 and 1, with scores of 0.18 for phobic anxiety and 0.36 for psychosis. Furthermore, another study (21) showed that the scores of all items in the Vietnamese population ranged from 0.3 to 0.8, and the scores did not markedly change over time. A previous study (22) compared sample populations from three different regions in Chile. In all three regions, obsessive-compulsive symptoms had the highest scores, and psychosis and phobia had the lowest scores. Another study (15) investigated students from two universities in Hungary and found that the various items of the SCL-90 had scores between 0.37 and 0.8; obsessive-compulsive symptoms had the highest scores, and psychosis had a lower score than paranoid ideation. Previous studies (23, 24) have found that the SCL-90 scores of Danes were higher than those of the US population, but the difference was <1 point. The above overall scores were all lower than those in 2019 in China, but they are highly consistent. For example, the score of obsessive-compulsive symptoms is consistently the highest, the score of psychosis is lower than that of paranoid ideation, and the scores of phobic anxiety and psychosis are the lowest. According to the data from these countries, the overall trend in the SCL-90 scores remains the same in different countries. However, the overall score is relatively high in China possibly due to factors related to cultural and economic development. This finding indicates that the SCL-90 should have different evaluation criteria in different countries.

Because the sample populations used in this study were regional, there may be some differences from the norms applicable to China as a whole, and some errors are expected. Further studies will include sample populations from more regions for further analysis and research.

## Data Availability Statement

All datasets generated for this study are included in the article/supplementary material.

## Ethics Statement

The studies involving human participants were reviewed and approved by the ethics committee/IRB of Tianjin Ciming checkup center.

## Author Contributions

WDa, YX, WDo, XY, and YM contributed to the conception and design of the study. JL and JJ organized the database. JJ and YM performed the statistical analysis. WDa and YX wrote the first draft of the manuscript. YM, SZ, and WW wrote sections of the manuscript. All authors contributed to manuscript revision and read and approved the submitted version.

## Conflict of Interest

JJ, BY, JL, CF and HY are employees of Beijing Wanling Pangu Science and Technology Ltd. The remaining authors declare that the research was conducted in the absence of any commercial or financial relationships that could be construed as a potential conflict of interest.
